# Understanding the Influence of Web-Based Information, Misinformation, Disinformation, and Reinformation on COVID-19 Vaccine Acceptance: Protocol for a Multicomponent Study

**DOI:** 10.2196/41012

**Published:** 2022-10-17

**Authors:** Eve Dubé, Shannon E MacDonald, Terra Manca, Julie A Bettinger, S Michelle Driedger, Janice Graham, Devon Greyson, Noni E MacDonald, Samantha Meyer, Geneviève Roch, Maryline Vivion, Laura Aylsworth, Holly O Witteman, Félix Gélinas-Gascon, Lucas Marques Sathler Guimaraes, Hina Hakim, Dominique Gagnon, Benoît Béchard, Julie A Gramaccia, Richard Khoury, Sébastien Tremblay

**Affiliations:** 1 Department of Anthropology Laval University Quebec, QC Canada; 2 Faculty of Nursing University of Alberta Edmonton, AB Canada; 3 Vaccine Evaluation Center BC Children’s Hospital Research Institute University of British Columbia Vancouver, BC Canada; 4 Department of Community Health Sciences University of Manitoba Winnipeg, MB Canada; 5 Department of Pediatrics Dalhousie University Halifax, NS Canada; 6 School of Population and Public Health University of British Columbia Vancouver, BC Canada; 7 School of Public Health Sciences University of Waterloo Waterloo, ON Canada; 8 Faculty of Nursing Laval University Quebec, QC Canada; 9 Department of Social and Preventive Medicine Laval University Quebec, QC Canada; 10 Department of Family and Emergency Medicine Laval University Quebec, QC Canada; 11 Department of Computer Science and Software Engineering Laval University Quebec, QC Canada; 12 Department of Biohazard Quebec National Institute of Public Health Quebec, QC Canada; 13 School of Psychology Laval University Quebec, QC Canada

**Keywords:** vaccine hesitancy, COVID-19, misinformation, vaccine decisions, disinformation, online, vaccine, vaccination

## Abstract

**Background:**

The COVID-19 pandemic has generated an explosion in the amount of information shared on the internet, including false and misleading information on SARS-CoV-2 and recommended protective behaviors. Prior to the pandemic, web-based misinformation and disinformation were already identified as having an impact on people’s decision to refuse or delay recommended vaccination for themselves or their children.

**Objective:**

The overall aims of our study are to better understand the influence of web-based misinformation and disinformation on COVID-19 vaccine decisions and investigate potential solutions to reduce the impact of web-based misinformation and disinformation about vaccines.

**Methods:**

Based on different research approaches, the study will involve (1) the use of artificial intelligence techniques, (2) a web-based survey, (3) interviews, and (4) a scoping review and an environmental scan of the literature.

**Results:**

As of September 1, 2022, data collection has been completed for all objectives. The analysis is being conducted, and results should be disseminated in the upcoming months.

**Conclusions:**

The findings from this study will help with understanding the underlying determinants of vaccine hesitancy among Canadian individuals and identifying effective, tailored interventions to improve vaccine acceptance among them.

**International Registered Report Identifier (IRRID):**

DERR1-10.2196/41012

## Introduction

### Background

Prior to the pandemic, web-based misinformation and disinformation were identified as key issues that negatively impact vaccine acceptance [1,2]. The COVID-19 pandemic has heightened these issues to a point where the World Health Organization director noted that the world was not just fighting a pandemic but also an infodemic [3]. For example, *reinformation* [4] is a form of disinformation that could have contributed to COVID-19 vaccine hesitancy [5]. Hyper-partisan news is not false per se—the events reported may be real—but their claim to be informative conceals the intention to manipulate readers into adopting the organization’s viewpoints [6,7]. For example, in Canada, the Rebel News and Global Research media outlets publish controversial news, and their coverage is often qualified as misleading [8]. Fact-checking devices are already being used in journalism, policy making, and education to limit the detrimental effects of disinformation. However, checking facts has proven to be insufficient for countering reinformation and is less efficient with information that is not false per se but is biased and emotionally loaded in its presentation. Textbox 1 presents the definitions of some of the key concepts used in our study.

**Definitions of key concepts.**

**Key concepts**

- Infodemic: overabundance of information—true, false or misleading—that makes it harder for people to know what to do [9]
- Misinformation: false information that is not created with the intention of causing harm [10]
- Disinformation: false information that is deliberately created to cause harm [10]
- Malinformation: information that is based on reality and used to inflict harm [10]
- Reinformation: hyper-partisan information created by groups that are self-proclaimed alternative news organizations [4]

The COVID-19 vaccination campaign is unprecedented not only in terms of scale and the public’s attention toward the safety and effectiveness of the different vaccines but also in terms of misinformation and disinformation about COVID-19 vaccination, which were already prominent even before the first vaccines were approved for use [11,12]. In Canada, high rates of COVID-19 vaccine uptake were reached overall in adults, but these rates have been lower among equity-deserving groups, such as racialized people, newcomers, and Indigenous people [13,14]. Moreover, among those who accepted initial doses, there is lower uptake or willingness with regard to completing their initial series of vaccines or accepting additional or booster doses [15]. Studies have shown that parents, even those who are vaccinated themselves, are more hesitant toward vaccinating their children [16,17].

Experts often attribute lower than expected vaccine uptake rates to the negative impact of false or antivaccine information shared on the internet [18]. However, the role that web-based misinformation and disinformation play in individual and community COVID-19 vaccine decision-making in real-life settings remains poorly understood, particularly among equity-deserving groups. Most studies are descriptive (ie, content analyses of antivaccine websites and social media) or have tested the impact of experimentally created fictitious websites [1,19,20], leaving important questions unanswered. For example, there is little known on the influence of the writing style of alleged facts about COVID-19 vaccination or the characteristics of web-based content on people’s attitudes toward COVID-19 vaccines. It is unclear if information-seeking practices differ between vaccine-hesitant parents and vaccine-confident parents and to what extent vaccine-hesitant parents are being led into echo chambers by social media algorithms. The consequences of experiences of inequity and systemic racism within the health system on trust in official sources of COVID-19 vaccine information (eg, governments and public health or health systems) remain unclear. Finally, research into interventions to address misinformation and disinformation is growing rapidly, but there is a need to identify effective interventions that could be easily and rapidly implemented within public health practices to reduce the impact of misinformation and disinformation on vaccine acceptance [21].

### Objective

The overall aims of our study are to better understand the influence of web-based misinformation and disinformation on COVID-19 vaccine decisions and investigate potential solutions to reduce the impact of web-based misinformation and disinformation about vaccines.

Specifically, the study has the following four objectives: (1) describe the infodemic and web-based discourses related to the generation and spread of misinformation and disinformation on COVID-19 vaccines in Canada by evaluating the quality of content with presumed journalistic value in the digital environment and modeling the different characteristics of social network conversations following COVID-19 news items; (2) examine the impact of web-based misinformation and disinformation and the infodemic on COVID-19 vaccine decisions by using a web-based randomized controlled experimental survey; (3) explore attitudes, values, risk perceptions, beliefs, behaviors, and information seeking about COVID-19 vaccination in an ethnically diverse sample of vaccine-hesitant, Canadian parents of children aged 12 to 17 years; and (4) investigate potential solutions to address COVID-19 vaccine hesitancy in Canada and reduce the impact of web-based misinformation and disinformation about vaccines by reviewing gamified digital tools for enhancing vaccine acceptance and uptake.

## Methods

This is a protocol for a multicomponent study that will involve several research approaches. Each objective’s methodological approach is described below.

### Understanding the Potential Impact of Web-Based Misinformation and Disinformation on Vaccine Acceptance and Their Characteristics

Objective 1 is concerned with the *production sphere of reinformation news*. A machine learning algorithm will be trained to identify and detect reinformation content about COVID-19. The writing styles of mainstream news articles will be assessed to determine whether the nature of neutral and objective storytelling is in line with traditional media guidelines. This will include an assessment of visual (eg, the layout of a page), linguistic (eg, the choice of words), narrative, journalistic (eg, identifying fundamental questions that every news article is supposed to answer), and structural characteristics (eg, the type of content goes in the initial, middle, or final paragraphs) [22]. The procedure for analyzing mainstream media news articles will be repeated on alternative media websites to determine their enunciative and storytelling characteristics and identify how they differ from traditional news. In addition, we are developing a corpus of real and reinformation news from more than 55,000 web-based news and reinformation articles. This corpus will be used to train algorithms to rank the quality of news articles based on their style. Objective 1 also focuses on social media that allow readers to submit comments and express their viewpoints on news articles, providing a window into audiences’ reception of content in a more dialogic way. The stylistic attributes, semantic attributes, and meta-attributes of the messages will be identified via a machine learning algorithm to study the course of conversations following news about COVID-19 vaccines. These attributes will then be used to define classes of messages (eg, comments, jokes, questions, answers, and attacks). We will also train a hidden Markov model on social media conversations to discover their flows and impacts on readership and identify critical messages that may affect a conversation in different ways.

**Figure 1:**
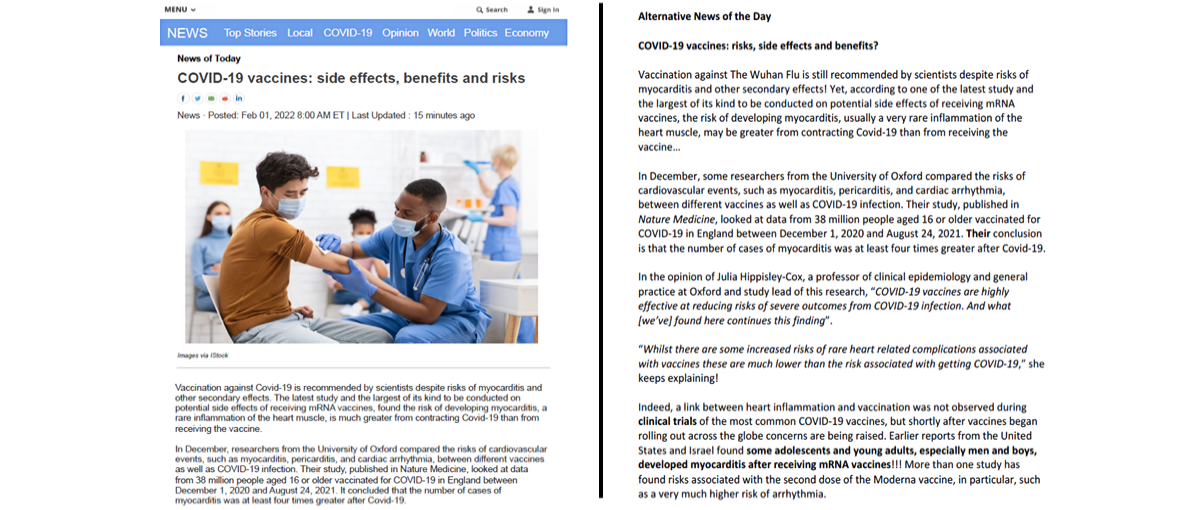
Examples of news article 1 (journalistic style and journalistic layout) and news article 4 (ideologically biased style without a journalistic layout). mRNA: messenger RNA.

Objective 2 focuses explicitly on *audiences of reinformation*. The aim is to better understand how readers perceive information about COVID-19 based on the writing style in which such content is conveyed. In an infodemic context where the quality of information available about the COVID-19 pandemic varies across media, the enunciation of discourse may have a negative effect on readers' attitudes toward vaccination against COVID-19. Accordingly, one strategy behind reinformation and disinformation is to mimic traditional media writing styles while rejecting traditional media [23]. However, there is evidence that readers who perceive information as tentative often rate such information as less credible [24]. Using the Qualtrics panel system (Qualtrics International Inc), we will conduct a web-based survey (n=500) to distinguish the effect of ideologically biased material from that of journalistic style–based material. In collaboration with a journalist from a major Canadian Anglophone newspaper, we developed a news article on the potential side effects of vaccination against COVID-19. The news article that we developed will serve as a basis for comparing different styles (ie, journalistic style–based material vs ideologically biased material) and visual layouts (ie, journalistic layout with colored graphs vs nonjournalistic layout) within a 2-by-2 factorial (between-group) design (Figure 1).

In news articles 2 (ideologically biased style and journalistic layout) and 4 (ideologically biased style without a journalistic layout), the original text from a media story was edited to match modalities that bias the style of text [25]. For example, if terms such as *Wuhan flu*, instead of *COVID-19*, are used, the ideology and attitude of the information provider is encoded and may be shared by and to readers. The use of an ideologically biased style could be one of the drivers of vaccine hesitancy.

### Exploring the Role of Web-Based Misinformation and Disinformation About COVID-19 in Parental Vaccine Hesitancy

With objective 3, we aim to gain a better understanding of the factors that result in COVID-19 vaccine hesitancy, including the potential influence of web-based content and other information sources. We will conduct semistructured interviews with an ethnically diverse sample of Canadian parents of children aged 12 to 17 years (n=50). We will focus on adolescent vaccination, as the COVID-19 vaccine uptake rate of 12- to 17-year-olds in Canada is among the lowest in the country [15]. Previous studies have also shown that parents can make vaccination decisions for their children that are different from those they make for themselves [16,26]. Although Canadian adolescents can provide consent for vaccination (the age of medical consent ranges from 14 to 16 years in some provinces, while others have not set any specific age), many studies have shown that these decisions are often aligned with parental views and values [27,28]. A better understanding of the reasons why parents hesitate to accept a full course of vaccines for their children can provide a basis for the development of public health interventions, as these parents’ attitudes may be more amenable to change than the attitudes of those who are strongly opposed to vaccination for themselves and their children. The recruitment of parents will be facilitated through previous surveys by our research team, in which some participants agreed to be contacted for subsequent qualitative studies. Two pan-Canadian surveys were conducted among the general public and equity-deserving groups (ie, racialized people, newcomers, Indigenous people, and persons whose first language is not English or French) within Canada in December 2020 and in October and November 2021 [29]. We will use the results of the latest survey to identify parents who were unsure about having, or were unwilling to have, their 12- to 17-year-old children vaccinated against COVID-19 and invite them to participate in individual interviews. We will also use sociodemographic information, including gender, location, age, and education status, to ensure that we recruit a diverse sample of vaccine-hesitant parents. This purposive recruitment will allow us to explore how social location affects vaccine hesitancy. The interviews will elicit information from the parents about rationales behind COVID-19 vaccination decisions and hesitation for themselves and their adolescents, including the extent to which participants feel that web-based information has influenced their decisions about COVID-19 vaccination. The interviews will be conducted in English or French and transcribed verbatim. A thematic analysis will be performed with NVivo software (QSR International). The interviews will allow us to situate the findings for objectives 1 and 2 in the real world of local knowledge systems (vaccine stories and experiences) that are used by diverse, vaccine-hesitant parents.

### Identifying Potential Web-Based Solutions to Counter Misinformation and Disinformation About Vaccines

Although it is often suggested that web-based misinformation and disinformation about vaccines negatively influence vaccine acceptance and uptake, very few web-based interventions that promote vaccination have been shown to be effective [30]. Previous reviews suggested that gamification can have positive effects on health-related behaviors and their determinants and may be a promising vehicle for inoculating the public against misinformation and disinformation, but limited data exist with regard to applying gaming interventions for vaccination [31]. With objective 4, we aim to review the existing, gamified, digital tools that have been implemented or evaluated across various populations and encourage vaccination uptake. We will conduct a scoping review and environmental scan, using relevant keywords in 9 databases and on Google. Individual interviews with experts in the field (eg, game developers and experts in gamification and health behaviors) will be conducted to complement the web-based searches and identify other tools. We will undertake a content analysis to assess the gamification elements and modalities and behavior change techniques that were used in the tools [32]. More information on this objective methodology is available on the Center for Open Science website [33]. We will triangulate this analysis with the findings of the other parts of our study to explore whether the content and writing style of games are likely to positively influence peoples’ views and attitudes toward vaccines (identified via objectives 1 and 2) and identify which of these games are aligned with the information needs and preferences of vaccine-hesitant parents (identified via objective 3).

### Ethics Approval

The study was approved by the Research Ethics Committee of CHU de Québec-Université Laval. Participants’ data will be stored on secure servers.

## Results

As of writing this paper (September 1, 2022), data collection has been completed. The research team is performing quantitative and qualitative analyses. The dissemination of findings and conclusions through scientific papers and conference abstracts will occur in the upcoming months.

## Discussion

Although the scientific consensus on the public health benefits of vaccination is unequivocal, there is no such agreement on how best to address vaccine hesitancy and combat web-based misinformation and disinformation about COVID-19 vaccines. Our study relies on an interdisciplinary team of researchers with extensive research expertise in understanding vaccine decision-making in Canada [34-37]. Our previous work has shown that technical, psychological, cultural, and societal factors can affect vaccine decision-making [34,35], and education interventions or information-based interventions for promoting vaccine acceptance can be unsuccessful if they are not grounded in the multiple ways in which knowledge is shared and heard within the communities of our increasingly interconnected world [38-40] Vaccine acceptance requires the public’s trust in health care providers, public health agencies, and health systems, which play a critical role in both communicating accurate information and dispelling misinformation and disinformation. Our study will contribute to the development of tailored strategies that are tested, are informed by evidence, and take into account the complex and context-specific nature of vaccine acceptance [41,42].

This protocol presents the methods that we will apply to better understand the influence of web-based information on COVID-19 vaccine decisions. The findings of our study will contribute to a better understanding of how people use current web functionalities, how such usage influences expectations about information sources and vaccination decision-making processes, and the implications for health authorities’ communication strategies [43]. As additional doses of COVID-19 vaccines are recommended, our study will identify promising solutions to address the influence of misinformation and disinformation regarding vaccines. In the current infodemic context, our study will identify tools and solutions that align with how people access and use information in their vaccination decision-making processes. Given the amount of financial and human resources that are invested in developing and diffusing communication materials about vaccination, it is critical to understand how to optimize these tools to ensure that they work as intended.
